# Construction of Lambda-Cyhalothrin Nano-Delivery System with a High Loading Content and Controlled-Release Property

**DOI:** 10.3390/nano8121016

**Published:** 2018-12-06

**Authors:** Yue Shen, Huaxin Zhu, Jianxia Cui, Anqi Wang, Xiang Zhao, Bo Cui, Yan Wang, Haixin Cui

**Affiliations:** Institute of Environment and Sustainable Development in Agriculture, Chinese Academy of Agricultural Sciences, Beijing 100081, China; shenyue@caas.cn (Y.S.); 82101172075@caas.cn (H.Z.); 82101176075@caas.cn (J.C.); angelking521@163.com (A.W.); zhaoxiang@caas.cn (X.Z.); cuibo@caas.cn (B.C.)

**Keywords:** Lambda-cyhalothrin, solvent evaporation method, nanoparticles, foliage adhesion

## Abstract

Traditional pesticide formulations are limited by large organic solvent consumption, poor dispersibility, and poor foliar adhesion, resulting in low effective pesticides utilization and environmental pollution. To prolong the foliar pesticide retention and release time, a high lambda-cyhalothrin (LC)-loaded nano-delivery system was constructed, using polylactic acid (PLA) as a carrier through a solvent evaporation method. The obtained results showed that the stabilizer concentration, water–oil ratio, and carrier content exert a major influence on the LC loading, particle size, and size distribution. The prepared LC/PLA nanoparticles have a uniform spherical shape with a smooth surface. The size of the nanoparticles was less than 200 nm, and the LC loading capacity reached up to 46.6 wt.%, with a high encapsulation efficiency (exceeding 90%). Adjustment of the shear and ultrasonic time changed the size of the nanoparticles. Significant differences were found in the sustained release properties of LC/PLA nanoparticles with different LC loadings. The foliage adhesion of the LC nano-delivery system far exceeded that of the commercial LC formulation due to a low surface tension and a low contact angle, this foliage adhesion would greatly help to improve pesticide utilization.

## 1. Introduction

The development of both efficient and safe green pesticides is a major strategic goal to ensure sufficient food production and ecological security. It is of great importance to alleviate current pesticide residues and environmental pollution, as well as to promote a sustainable development of the pesticide industry [1]. Most of the active ingredients of existing pesticides are high-activity, water-insoluble organic compounds. It is usually necessary to add auxiliary ingredients such as carriers, solvents, emulsifiers, and dispersing agents to prepare various types of formulations [2]. Therefore, using advanced carrier materials and loading methods to improve the effectiveness and utilization of pesticides, as well as reducing the residual pollution in non-target areas and environments, has become a scientific problem in modern agriculture that needs to be urgently solved.

Recently, the rapid development of nanotechnology has provided a new scientific methodology for modern agricultural science. In particular, the use of nanomaterials for the construction of a pesticide nano-delivery system to transfer and release functional molecules to target sites has become a current research hotspot in the field of nanoscience and pesticide science [3,4]. Nanomaterials are used to load pesticide molecules via both adsorption and encapsulation [5]. Consequently, the function of pesticide formulation will be improved as follows: (1) promoting the dispersibility and stability of poorly soluble pesticides in water; (2) promoting the target absorption, thus enhancing the biological activity; (3) promoting the adhesion and permeability of pesticide-loaded particles on crop surfaces, as well as promoting the targeted transfer and enrichment of pesticide active ingredients to target sites such as pests and pathogens, reduce shedding and loss, and improve effective utilization rate; (4) accurately controlling the pesticide release rate and maintaining minimum effective utilization, in addition to reducing the loss of efficacy as a result of loss, drift, and decomposition [6,7,8]. 

Lambda-cyhalothrin (LC) is a highly efficient insecticide with a broad insecticidal spectrum, as well as strong contact, and stomach toxicity. It achieves a good control effect on lepidopteran pests, but easily becomes ineffective due to the development of resistance in response to long-term use. Meanwhile, skin allergies caused by LC have affected the market for product sales. Accordingly, in recent years, several materials such as SiO_2_, alginate derivatives, and lignin have been used as carriers to develop the LC-loaded slow release systems [9,10,11]. Although these LC-loaded systems showed certain stability and controlled-release properties, most of them were prepared by relatively complex techniques and were difficultly scalable. In addition to these techniques, polylactic acid (PLA), which have been approved by the Food and Drug Administration (FDA) and the European Medicines Agency, have been extensively investigated as drug carriers for the sustained and controlled release in forms of microspheres/nanoparticles [12,13]. At present, the most mature process for preparing PLA drug-loaded microspheres/nanoparticles is the solvent evaporation method, which can be further divided into oil-in-water (O/W) emulsion and water-in-oil-in-water (W/O/W) double emulsions [14,15]. To date, the construction of LC-loaded PLA nanoparticles has not received significant efforts, neither has the optimization of the process conditions.

In this work, a high LC-loaded nano-delivery system was successfully constructed. The influence on LC loading, particle size, and dispersibility of different process conditions including organic solvents, stabilizer concentration, water–oil ratio, and carrier content were systematically investigated. The LC controlled release behavior, surface properties of the formulation (contact angle and surface tension), stability (photoresistance and storage stability), as well as the adhesive property on crop foliage were further investigated. 

## 2. Materials and Methods 

### 2.1. Materials

The polylactide (PLA) and LC (97%) were supplied by Nature Works Co. (Minnetonka, MN, USA) and Jinyue Biotechology Co. Ltd. (Beijing, China), respectively. The polyvinyl alcohol (PVA), 87–90% hydrolyzed with an average *M_w_* of 30,000-70,000, was supplied by Sigma-Aldrich Shanghai Trading Co., Ltd. (Shanghai, China). The dichloromethane (DCM) was provided by the Beijing Chemical Reagents Company, China. The chromatography grade of methanol and acetonitrile were purchased from Fisher (Beijing, China) and used for high-performance liquid chromatography (HPLC). The water was purified by a Milli-Q water purification system and used in all experiments.

### 2.2. Preparation of the LC Nano-Delivery System

The quantitative ratios of both LC and polylactic acid were dissolved in DCM as an oil phase; the aqueous phase was a 2% PVA aqueous solution. The oil phase was added dropwise into the aqueous phase. The mixture was sheared at a power of 13,000 rpm for 10 min to prepare a coarse emulsion. Subsequently, to reduce the size of the emulsion, the coarse emulsion was immediately transferred to an ultrasonic homogenizing unit (JY90-IIN, 1800W power, 80% amplitude with 5 s pulses). The emulsion was then stirred overnight at room temperature under magnetic stirring (500 rpm) for the evaporation of the residual organic solvent. The prepared nanopesticide system was collected by centrifugation, washed three times with distilled water, and then freeze-dried to obtain a free-flowing powder.

### 2.3. Characterization

The ingredients of the LC/PLA nanoparticles (NPs) were qualitatively determined via fourier transform infrared spectrometer (FTIR, Nicolet IS10, Thermo Fisher Scientific, Waltham, MA, USA). The mean diameter and polydispersity index (PDI) of the LC/PLA NPs were measured via dynamic light scattering (DLS) technique with a Zetasizer Nano ZS90 (Malvern Instruments, Malvern, UK) in a water solution. The morphology of the dried NPs was characterized using a scanning electron microscope (SEM, Hitachi SU8010, Hitachi Ltd., Tokyo, Japan) at an accelerating voltage of 5 kV. The structure of the dried NPs was characterized by transmission electron microscopy (TEM, Hitachi HT7700, Hitachi Ltd., Tokyo, Japan) at an accelerating voltage of 80 kV. The samples were obtained by spreading and drying the diluted system on carbon-coated copper grids. The surface tension of different formulations was directly tested on a surface tension meter (JK99BM, Powereach, China) at 25 °C. The contact angles were measured by a contact angle meter (JC2000D2M, Powereach, China) at 25 °C.

### 2.4. Determination of Loading and Encapsulation Efficiency

The pesticide loading capacity (*DL*) is defined as the mass percentage of the loaded LC to the total solids in the LC/PLA NPs. About 30 mg of the samples were weighed and dissolved in 50 mL of acetonitrile, and the mixture remained in a shaking tank overnight at a constant temperature to completely dissolve the carrier material. After the solution was filtered, the mass concentration of LC in acetonitrile was examined by HPLC (Agilent 1260 system with a WATO45905 C18 column) under a detection wavelength of 278 nm [16], and the LC loading capacity of the NPs was calculated according to Equation (1). The encapsulation efficiency (EE), defined as the mass percentage of the loaded LC to the total LC used in the preparation process, was calculated according to Equation (2).

(1) $$DL\;(\mathrm{wt}.\%)=\frac{\mathrm{Mass}\;\mathrm{of}\;\mathrm{loaded}\;\mathrm{LC}}{\mathrm{Mass}\;\mathrm{of}\;\mathrm{NPs}}\times 100$$

(2) $$EE\;(\mathrm{wt}.\%)=\frac{\mathrm{Mass}\;\mathrm{of}\;\mathrm{loaded}\;\mathrm{LC}}{\mathrm{Mass}\;\mathrm{of}\;\mathrm{LC}\;\mathrm{added}}\times 100$$

### 2.5. Controlled Release of LC

The release behavior from different types of LC/PLA NPs and the LC technical were evaluated as follows: 0.03 g of sample was first suspended in 10 mL of the methanol/H_2_O mixture (3:2, *v/v*) and charged into a dialysis bag where the molecular weight cut-off (MWCO) was 14000. The bag was then immersed in 90 mL of a methanol/H_2_O mixture (3:2, *v/v*) in conical flasks and shaken at a temperature of 25 °C. Finally, the mixtures were placed in incubators and constantly shaken at a speed of 100 rpm. During dialysis, 5 mL of the solution outside the dialysis bag was collected at specified time intervals, while 5 mL of fresh solvent was added instead. The LC concentration of the solution was measured via UV-Vis spectrophotometer (UV-2600, Shimadzu, Japan) at a wavelength of 278 nm, and the cumulative release rate of LC for each sample was calculated by the concentrations of LC dissolved in release medium at different times according to Equation (3).

(3) $$R\%=\frac{\left(V_{t}\times C_{t}+V\times \sum C\right)}{W\times DL}\times 100$$

where *R*% represents the cumulative release rate, *V_t_* represents the volume of methanol/H_2_O mixture, *C_t_* represents the LC concentration measured at different time intervals, *V* represents the sampling volume, ∑C represents the concentration of continuous sampling, *W* represents the mass of the NPs, and *DL* represents the LC loading capacity.

### 2.6. Stability Studies

The UV-shielding properties of the LC/PLA NPs were tested with the technical LC as a control. In detail, the NPs were dispersed and equally divided into culture dishes, which were illuminated under a UV lamp (500 W) with a maximum emitting light of 365 nm wavelength at 25 °C. At specified time intervals (12, 24, 36, 48, 60, and 72 h), the samples were taken out of the reactor and the LC concentrations of samples were analyzed to calculate the photogradation rate.

## 3. Results and Discussion

### 3.1. Effects of Process Parameters on Size, Size Distribution, and Pesticide Loading Capacity of LC-Loaded Nanoparticles

#### 3.1.1. Effects of Organic Solvents

The most used method for preparing PLA microspheres is a one-step O/W emulsion solvent evaporation method [17,18,19]. Commonly used volatile solvents are slightly soluble in water with a solubility below 10% and a boiling point below 100 °C. Generally, dichloromethane, chloroform, acetone, benzene, and diethyl ether are used as solvents [20]. Since acetone is miscible with water, it is not suitable for the solvent evaporation method. Diethyl ether is also not suitable as a solvent because it is hardly soluble in water. Benzene is unsuitable as a solvent for this process due to its high toxicity and boiling point. Compared to these three common solvents, dichloromethane has the lowest water solubility and the appropriate boiling point, making it to be considered as the most ideal solvent. Therefore, dichloromethane was selected as the organic phase in this experiment.

#### 3.1.2. Effects of PVA Concentration in the External Water Phase

**Figure 1:**
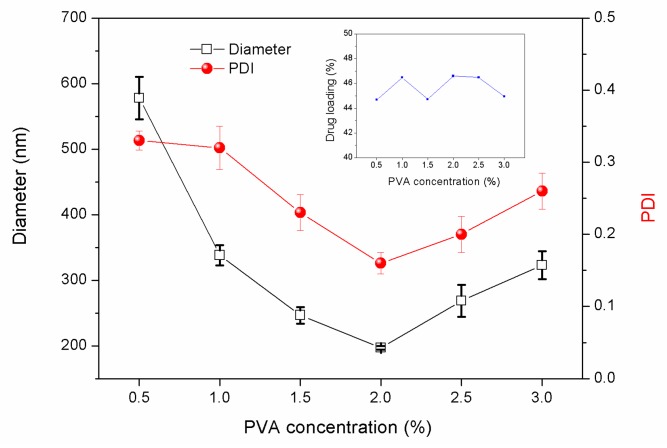
Particle size, size distribution, and LC loading capacities of PLA nanoparticles prepared under different PVA concentrations (water–oil ratio was 4:3, PLA concentration was 133 g/L, and LC-PLA ratio was 1:1).

**Figure 2:**
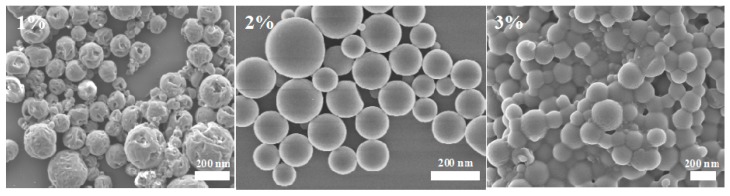
SEM micrographs of PLA nanoparticles prepared under different PVA concentrations (1%, 2%, and 3%).

**Figure 3:**
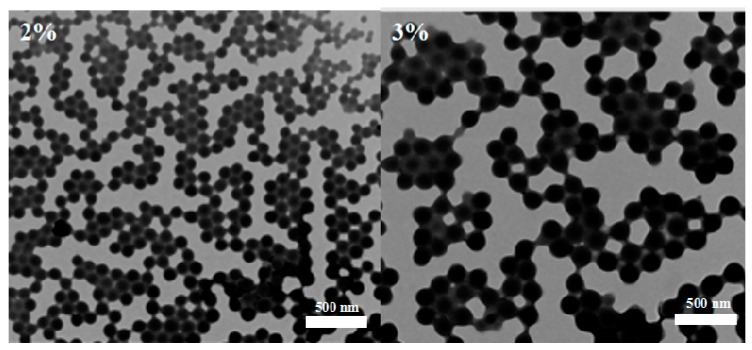
TEM micrographs of PLA nanoparticles prepared under different PVA concentrations (2% and 3%).

Polyvinyl alcohol (PVA), gelatin, hydroxypropylmethylcellulose (HPMC), and Tween are often used as emulsifiers for the solvent evaporation method [21]. The type, concentration, and emulsification time of the emulsifier are closely related to the size of the formed droplets, and directly affecting the quality of the microspheres. The results show that when the concentration of emulsifier PVA is low, both the sphericity and dispersibility of microspheres are poor, while the nanoparticle size is large (Figure 1). With the increase of PVA concentration, the sphericity and dispersibility of nanoparticles improve and the particle size decreases accordingly (Figure 2). With increasing PVA concentration, a higher viscosity leads to an easier nanoparticles formation, and the surface energy of the aqueous phase decreases. Thus, the surface energy difference between the organic phase and the water phase increases, the barrier effect is obvious, and the size of the nanoparticles tends to decrease [22,23,24]. However, when the PVA concentration is too high, the microsphere surface is not smooth and the particle size increases (Figure 2 and Figure 3). The LC loading amount essentially remains stable. For a PVA concentration of 2%, the LC loading amount was the largest, being 46.6%. In addition, the PDI value was the lowest, less than 0.1. The encapsulation efficiency exceeded 90%.

#### 3.1.3. Effects of the Water/Oil Phase Ratio

**Figure 4:**
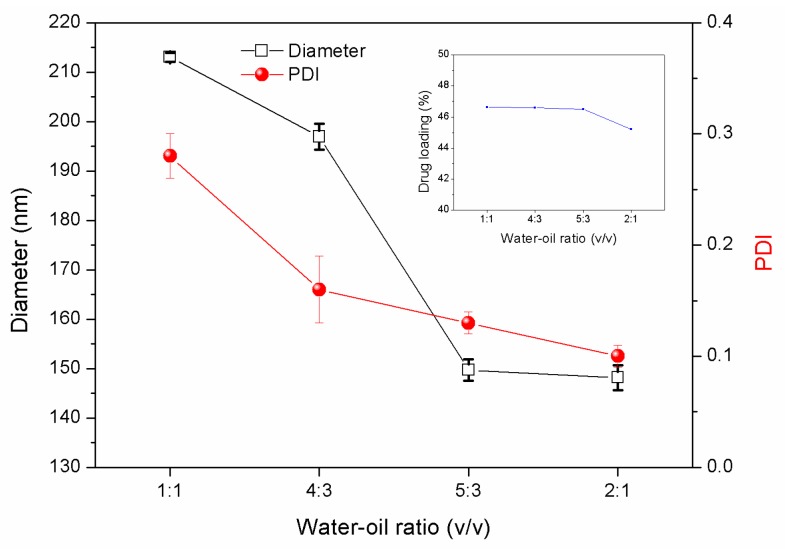
Particle size, size distributions, and LC loading capacities of PLA nanoparticles prepared under different water–oil ratios (PVA concentration was 2%, PLA concentration was 133 g/L, and LC-PLA ratio was 1:1).

To study the optimized conditions for the preparation of LC/PLA NPs, other factors influencing the characteristics of nanoparticles were optimized. The ratio of the water/oil phase has a great influence on particle size and uniformity [25]. As indicated in Figure 4, with the increase of the water–oil ratio, the sphericity gradually improves, the particle size decreases, and the dispersibility improves. This is because a higher water–oil ratio leads to a higher stabilizer content and consequently, the solution can be fully emulsified. Furthermore, not only does PVA have good hydrophilicity, but it also has the properties of having increased bioadhesiveness. It can penetrate into the polylactic acid molecule, and the hydrophobic segment can penetrate into the organic solvent, thus leading to the uniform droplet of the synthetic emulsion [26]. The prepared PLA NPs have a uniform spherical shape with a smooth surface.

#### 3.1.4. Effects of PLA Concentration

**Figure 5:**
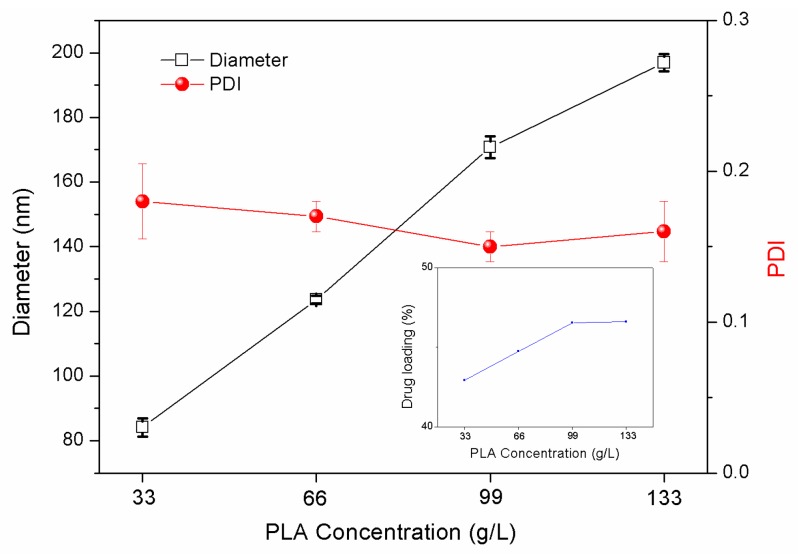
Particle size, size distributions, and LC loading capacities of PLA nanoparticles prepared under different PLA concentrations (PVA concentration was 2%, water–oil ratio was 4:3, and LC-PLA ratio was 1:1).

Particle size, size distributions, and LC loading capacities of LC/PLA nanoparticles as a function of PLA concentration are shown in Figure 5. The results show that the particle size gradually increases as the concentration of the PLA carrier increases. There is no significant change in polydispersity. Furthermore, as the concentration of the carrier increases, the LC loading of the nanoparticles also increases simultaneously. This is because a higher concentration of PLA leads to a greater viscosity of the internal dispersed phase, and an increased difficulty for the droplets to disperse under the same shear conditions. Moreover, since the amount of the stabilizer PVA remains constant, the barrier effect is decreased, allowing PLA to easily bond and form larger microspheres. However, if the concentration of PLA is too low, the nanoparticles will easily collapse from the inside. This can be attributed to the volatilization of the solvent during the curing process and will make the formation of smooth particles difficult. 

### 3.2. Preparation of LC-Loaded Nanoparticles with Different Sizes and Pesticide Contents

#### 3.2.1. Effects of Shear and Ultrasonic Times

The size of the particles in this experiment was affected by both shear and ultrasonic times. The effects of different shear and ultrasonic times on the mean diameter, polydispersity, and LC loading capacity of PLA NPs are summarized in Table 1. Since the shear time is prolonged, and the shear force of the emulsion system is increased, the dispersion of the dispersed phase in the continuous phase improves. Thus, the oil droplets of the oil phase and the particle size of the formed NPs become smaller (entries 1–2). However, the PDI values were a little bit higher. When the ultrasonic time is extended, the particle size does not significantly change, but the polydispersity improves and the PDI value decreases (entry 3). The LC/PLA NPs obtained under such preparation conditions have the highest LC loading capacity (49.4%). If the ultrasonic time remains constant, the particle size does not significantly change since the shear time increases. Therefore, the optimal shear time is 10 min.

#### 3.2.2. Effects of the PLA/LC Ratio

**Figure 6:**
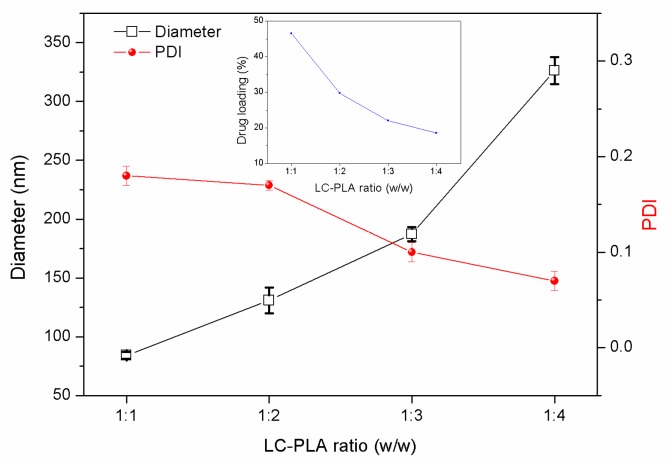
Particle size, size distributions, and LC loading capacities of PLA nanoparticles prepared under different LC-PLA ratios (PVA concentration was 2%, water–oil ratio was 4:3, PLA concentration was 133 g/L, and the amount of LC was constant).

The pesticide loading capacity of the NPs is one of the important factors for the regulation of the release rate within the delivery system [27]. Particle size, size distributions, and LC loading capacities of LC/PLA NPs as a function of the LC-PLA ratio are plotted in Figure 6. As the ratio of the LC to the PLA carrier increases, the particle size increases, the dispersibility improves, and the LC loading capacity increases. This is because the viscosity of the internal dispersed phase increases as the concentration of the PLA carrier increases. Under the same shear force, the internal dispersed phase is difficult to disperse, large droplets are easily formed, and the particle diameter increases. As shown in Figure 6, the measured LC contents were close to their theoretical values for all specimens, demonstrating that this method can effectively load LC into the PLA NPs. This may be attributable to the high loading efficiency and the poor aqueous solubility of LC, resulting in its low loss during the preparation process.

### 3.3. Controlled Release of LC

**Figure 7:**
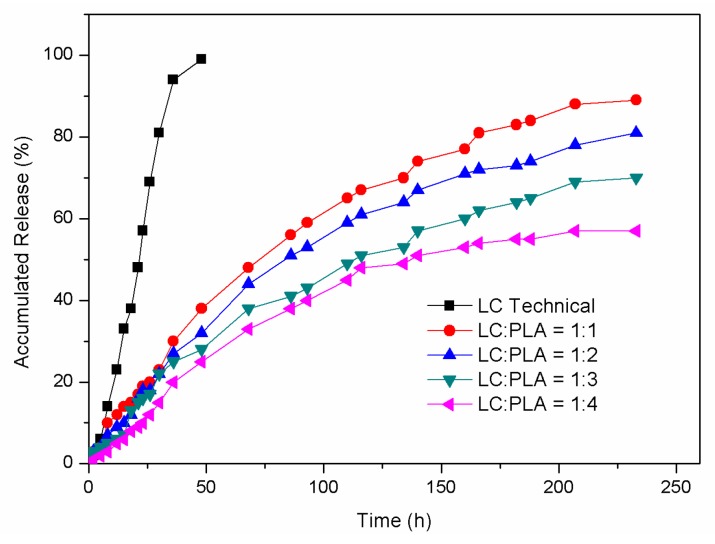
Release profiles of the technical LC and LC/PLA NPs with different LC loading capacities.

The release behaviors of LC from the LC/PLA NPs with different LC loading capacities were investigated in a methanol/H_2_O mixture (3:2, *v/v*) using the technical LC as control. Figure 7 shows the release profiles of the LC/PLA NPs and LC technical at room temperature. It should be noted that the release profiles did not represent the practical release behavior of LC/PLA NPs in an actual agricultural environment. The release rate of the LC will be slower in practical applications [15,16]. 

As shown in Figure 7, technical LC was completely released after 48 h. Compared to technical LC, the NPs released LC at relatively slow speeds and maintained its sustained release for longer periods. The accumulated release of LC from the LC/PLA NPs increased from 57% to 89% after 240 h, with an increase of LC loading capacity from 18.6% to 46.6% (LC:PLA from 1:4 to 1:1). This is because, as the ratio of the LC to the PLA carrier increases, the LC loading capacity increases, providing a greater concentration gradient of LC between the internal and external environment. Thus, the LC molecules dissolve and migrate to the surrounding more easily. 

**Figure 8:**
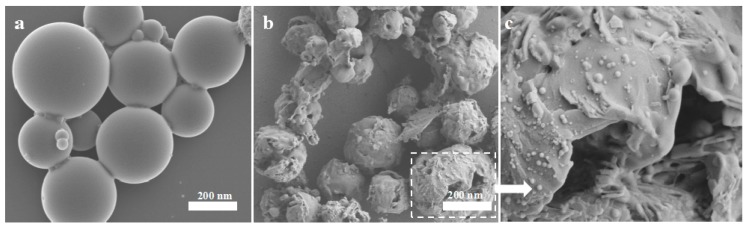
SEM micrographs of PLA nanoparticles (**a**) before and (**b**,**c**) after 10 days of pesticide release.

There are three modes in which the LC exists in the NPs: the LC can be adsorbed on the surface of the particles; the LC can be dispersed in the skeleton of the carrier material; and the LC can be wrapped inside the carrier material. From the release profile, the LC/PLA nano-delivery system has sustained release properties. The LC release profiles consisted of a relatively short burst release followed by a gradual release. During the early stage, the system mainly released pesticide on the surface of the NPs and in the shallow structure. After 10 days of release, collapsing and pores on the surface of the particles were observed (Figure 8), which were primarily caused by the decomposition of the carrier and the dissolution of the LC. The LC is also released by this dissolution and diffusion of the carrier.

### 3.4. Foliage Adhesion of the LC/PLA Nano-Delivery System

The effective utilization rate of pesticides sprayed on farmland is very low. Most of the liquids cannot be retained by the target plants, which leads to resources wastage and the environmental pollution. The actual utilization of biological target uptake is only less than 0.1% after dust drift and rainwater [28,29]. The control effect of pesticides is directly related to the wetting and retention ability of the formulation on the target plants, and the retention ability is related to the surface tension and the contact angle of the formulation on the leaf surfaces.

**Figure 9:**
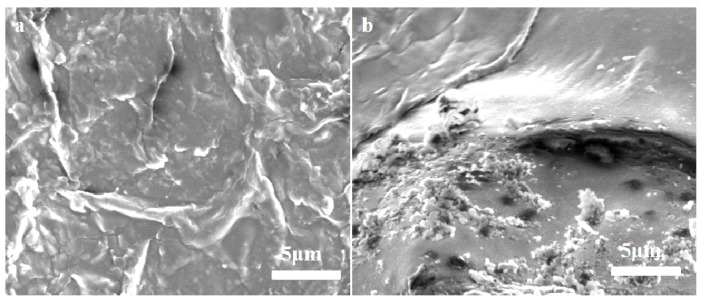
SEM images of (**a**) the LC/PLA nano-delivery system and (**b**) the commercial LC SC on cucumber leaves.

**Figure 10:**
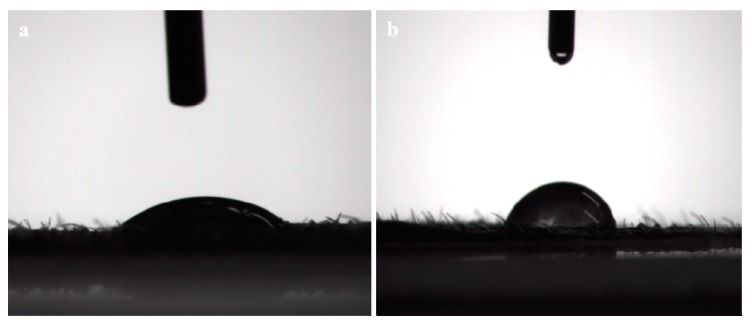
Contact angle of (**a**) the LC/PLA nano-delivery system and (**b**) the commercial LC SC on cucumber leaves.

The adhesion experiments were conducted to prove that the LC/PLA nano-delivery system has a better adhesion property on leaf surfaces than other commercial LC formulations, as shown in Figure 9. Compared to the LC suspension concentrate (SC), the LC/PLA nano-delivery system has a good dispersion property on cucumber leaves. The dried active ingredient in the LC nanoparticles was embedded between the veins due to their small size. Additionally, the test showed that the cucumber leaves had a high critical surface tension of 61.01 mN/m, and the surface tension of the prepared nanopesticide system was 55.63 mN/m. This was less than the critical surface tension of cucumber leaves, indicating that the system could wet and spread on cucumber leaves. The contact angles of different LC formulations were also investigated to evaluate their wetting ability. The results of this investigation are displayed in Figure 10. The commercial LC SC and LC/PLA nano-delivery system were 77.33° and 40.98°, respectively, on the cucumber leaves. These results clearly indicate that the LC/PLA nano-delivery system achieved stronger adhesion on crop leaves than the commercial LC formulation.

### 3.5. Stability Evaluation

**Figure 11:**
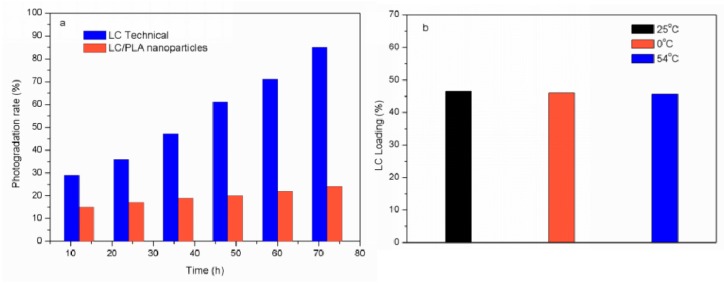
Comparison of (**a**) the LC photolysis percentage of the LC/PLA nanoparticles and the technical LC under UV irradiation, and (**b**) LC contents of the LC/PLA nanoparticles before and after 0 °C for 7 days and 54 °C for 14 days.

The photogradation of pesticides after spraying seriously disrupted the activities and reduced their utilization rates. Figure 11a shows the LC decomposition rate of the LC/PLA NPs and the technical LC under UV irradiation. The photolysis rate of technical LC exceeded 28% after 12 h of UV irradiation, while that of LC in the NPs was only 14%. Even after 72 h, only less than 20% of LC was degraded for the nanoparticles compared to up to 83% for the technical LC. The results clearly demonstrate that the LC/PLA NPs could effectively reduce LC photolysis, indicating that this system has the remarkable UV-shielding properties for LC. Measuring the LC content at different temperatures (25 °C, 0 °C, and 54 °C) also evaluated the storage stability of the NPs (Figure 11b). The LC content only showed negligible loss after storage at 0 °C for 7 days. A small LC loss was observed after 14 days at 54 °C due to the degradation of LC at high temperatures. When the pH value of the system was 7, the LC content remained unchanged after 7 days at room temperature. When the pH value of the system was 9, the LC content in technical LC was reduced by about 10% at room temperature. However, the LC content in LC/PLA nanoparticles remained stable. These results show that the LC/PLA NPs can be kept in a relatively stable state during storage. 

## 4. Conclusions

In summary, we successfully constructed a high LC-loaded nano-delivery system based on the solvent evaporation method in order to improve the stability and efficacy of LC. The processing parameters affecting LC loading, particle size, and dispersibility, which included organic solvents, stabilizer concentration, water–oil ratio, and carrier content, were systematically investigated. The size of LC/PLA NPs remained below 200 nm and the LC loading capacity reached up to 46.6 wt.% with a high encapsulation efficiency above 90%. The LC/PLA NPs possess sustained release properties. The LC release profiles consisted of a relatively short burst release followed by a gradual release. Moreover, the system had stronger adhesion on crop leaves than the commercial LC formulation, and could be kept in a relatively stable state during storage. As a green and efficient pesticide formulation, the LC/PLA nano-delivery system has great potential for further exploration.

## Figures and Tables

**Table 1:** Effects of shear and ultrasonic times on the size, size distribution, and LC loading capacity of LC/PLA nanoparticles ^a^.

Entry	Shear Time (min)	Ultrasonic Time (min)	Mean Diameter (nm)	PDI	LC Loading Capacity (%)
1	5	10	270.33 ± 5.13	0.17 ± 0.02	45.08
2	10	10	196.93 ± 2.66	0.16 ± 0.015	46.6
3	10	15	190.52 ± 3.74	0.10 ± 0.019	49.4
4	15	10	205 ± 1.39	0.11 ± 0.023	48.21

^a^ PVA concentration was 2%, water–oil ratio was 4:3, PLA concentration was 133 g/L, and LC-PLA ratio was 1:1.
